# Iliopsoas Impingement After Total Hip Arthroplasty: A Review of Diagnosis and Management

**DOI:** 10.7759/cureus.83391

**Published:** 2025-05-03

**Authors:** Zubair Younis, Muhammad A Hamid, Balu Ravi, Faliq Abdullah, Ahmed Al-Naseri, Khaldoun Bitar

**Affiliations:** 1 Orthopedics, Royal Wolverhampton NHS Trust, Wolverhampton, GBR; 2 Orthopedic Surgery, University Hospitals Birmingham, Birmingham, GBR; 3 Trauma and Orthopedics, Royal Wolverhampton NHS Trust, Wolverhampton, GBR; 4 Trauma and Orthopedics, Royal Shrewsbury Hospital, Shrewsbury, GBR

**Keywords:** acetabular component malposition, groin pain, hip flexor tendinitis, iliopsoas impingement, total hip arthroplasty

## Abstract

Iliopsoas impingement is a growingly acknowledged yet frequently overlooked cause of persistent groin pain after total hip arthroplasty (THA), occurring in a small percentage of patients undergoing the procedure. It typically results from mechanical irritation of the iliopsoas tendon by anterior acetabular component overhang, retained cement, long screws, or other prosthetic hardware. Clinically, patients report pain that worsens with active hip flexion, stair climbing, and transitioning from sitting to standing. Diagnosis involves a combination of clinical assessment, imaging techniques such as radiographs, CT scans, or ultrasound, and confirmatory image-guided diagnostic injections. Conservative management, including physiotherapy and corticosteroid injections, may offer temporary relief but is often insufficient for long-term resolution. Surgical options, particularly iliopsoas tenotomy or acetabular component revision, are indicated in refractory cases and have demonstrated high success rates with improved functional outcomes. Early recognition and appropriate intervention are critical for optimizing postoperative recovery and improving the quality of life in affected patients.

## Introduction and background

Ongoing discomfort following primary total hip arthroplasty (THA) is uncommon. However, when it does occur, it can stem from multiple factors, such as infection, implant instability, or bone loss around the prosthesis due to wear debris [1]. In some cases, pain in the hip or buttock may actually originate from spinal pathology or, less commonly, from abdominal or vascular conditions [2].

Iliopsoas tendinitis is a known cause of anterior hip pain, typically occurring when the tendon snaps over a bony prominence as the hip moves from flexion to extension [3]. After THA, iliopsoas tendinitis is often an overlooked but important cause of ongoing groin pain, occurring in about 4.3% of primary THA cases [4]. Patients usually experience pain that intensifies during physical activities, active hip flexion, climbing stairs, and actions like getting in and out of a car [1]. The onset of iliopsoas tendinitis in patients after THA is often associated with mechanical impingement due to improperly positioned acetabular components, leftover cement, overly long screws, or the presence of an acetabular cage or reinforcement ring [5,6]. Furthermore, factors such as excessive horizontal offset, increased leg length, or discrepancies in limb length can contribute to ongoing iliopsoas irritation [5]. In some cases, persistent iliopsoas tendinitis can occur even when no structural issue is identified [7].

Although iliopsoas tendinitis is frequently linked to overuse injuries in athletes, it is often not considered a source of pain after THA, resulting in delays in diagnosis and treatment [8]. Early identification and management are critical, as persistent iliopsoas tendinitis can significantly impair mobility, delay rehabilitation, and affect overall outcomes following hip replacement surgery. This article presents a narrative review of iliopsoas impingement following THA, discussing its anatomy, diagnosis, and management options.

## Review

Anatomy

The iliopsoas is a key muscle group in the hip area, comprising the psoas major and the iliacus. The psoas major originates from the lumbar vertebrae and extends to the lesser trochanter of the femur, while the iliacus arises from the iliac fossa and also inserts at the lesser trochanter. These muscles converge to form the iliopsoas muscle and tendon, which pass beneath the inguinal ligament to attach to the anteromedial lesser trochanter [9,10].

**Figure 1:**
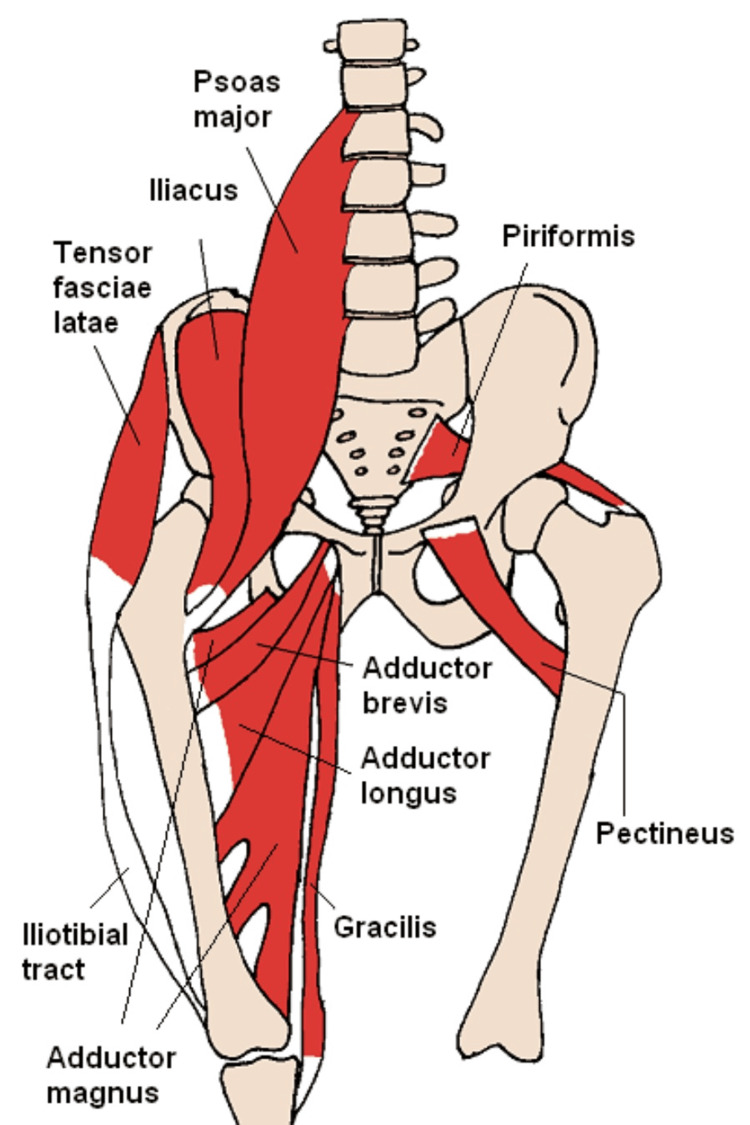
Schematic illustration of the iliopsoas anatomy and its relationship to the hip joint Image Credits: Wikipedia [12].

Anatomic studies have shown that the iliopsoas tendon complex is more complex than traditionally described, with two variations: one where the iliacus fibers stop at the femoral neck, and another where they continue to the lesser trochanter (Figure 1) [11]. The iliopsoas bursa separates the tendon from the hip capsule, and the tendon can become intra-articular if the anterior capsule is altered during THA [1]. 

Functionally, the iliopsoas is the main hip flexor and also aids in external rotation. The psoas major is innervated by the lumbar plexus (L1-L3), while the iliacus is supplied by the femoral nerve (L1-L3), with blood from the internal iliac artery. Together, these muscles stabilize the hip joint and are crucial for movement and posture [9,10]. 

Clinical presentation

Iliopsoas impingement can develop in one to 96 months after both primary and revision THA, regardless of whether a cemented or cementless acetabular component was used [5,13]. Patients typically experience persistent and severe groin pain, which is exacerbated by activities such as climbing stairs, getting in and out of bed, rising from a seated position, and entering or exiting a car [13]. In some cases, patients may require manual assistance to lift their limbs due to the severity of discomfort. A snapping or "clunking" sensation may also be reported [1]. 

During clinical examination, passive hip movement is typically pain-free, but active movement, particularly hip flexion, is restricted by pain [14]. While passive straight leg raises do not induce pain, active straight leg raises reproduce symptoms and are difficult to maintain due to pain. Physical examination may reveal subtle signs, such as a slight limp and tenderness in the groin area [14]. Although rare, a palpable snap might be detected, and in cases of significant bursitis, a palpable mass may be present. Typically, patients do not experience pain while walking, but pain can be triggered or intensified by resisted seated hip flexion or a straight leg raise [15]. Additionally, pain may occur with passive hyperextension and active external rotation and extension of the hip [4]. There is generally no evidence of significant leg length discrepancy, neurological abnormalities, or abductor muscle deficiency.

Diagnostic evaluation

**Figure 2:**
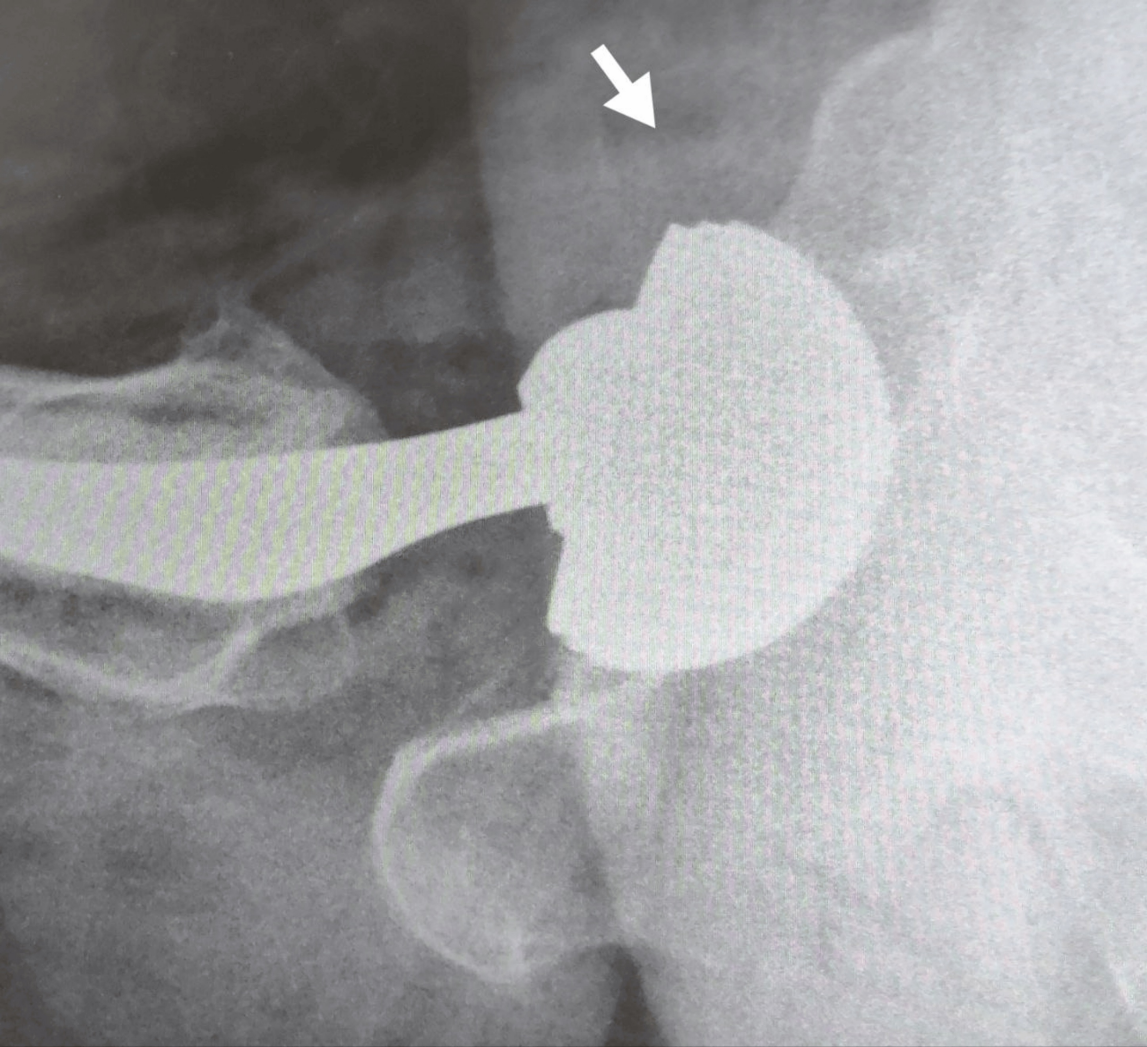
Cross-table radiograph with an arrow demonstrating anterior overhang of the acetabular component Image credit: Second author (consent obtained from the patient for use of the image in research).

The diagnostic evaluation of iliopsoas impingement as a cause of groin pain after total hip replacement begins with imaging studies. Standard anteroposterior pelvis, cross-table or true lateral, and frog-leg lateral radiographs are essential for assessing the acetabular component's position and identifying any changes (Figure 2). The cross-table or true lateral view is particularly useful for examining the anterior aspect of the acetabular component in relation to the anterior bony rim, which is crucial when considering iliopsoas impingement. If these radiographs are inconclusive, a CT scan can be employed to measure the acetabular component's version and detect any anterior overhang that could contribute to impingement. In cases where iliopsoas tendonitis is suspected, a diagnostic and therapeutic injection into the iliopsoas tendon sheath, guided by fluoroscopy, ultrasound, or CT, is recommended to confirm the diagnosis and provide symptom relief. If these tests do not reveal a definitive cause, further evaluation may include metal suppression MRI and serum metal ion levels to rule out other potential sources of pain. This targeted approach helps accurately diagnose iliopsoas impingement and differentiate it from other potential causes of groin pain following total hip replacement.

The initial focus should be to rule out common causes of persistent groin pain. Groin pain following THA can stem from a variety of intrinsic and extrinsic causes. The most common intrinsic causes include infection, aseptic loosening of the acetabular component, iliopsoas tendonitis, impingement, synovitis due to metal or polyethylene debris, pelvic osteolysis, and hidden acetabular or pelvic fractures [7]. Extrinsic causes involve pain from local neurological or vascular issues, inguinal hernia, metastatic cancer, and retroperitoneal pathology, as well as distant sources such as spinal pathology and radiculopathy [16]. If these are ruled out and iliopsoas impingement or tendinitis is suspected, further diagnostic evaluation is necessary. This involves imaging studies like plain radiography, CT, MRI, or ultrasonography, and a diagnostic injection test is often warranted [1].

Plain radiographs should include anteroposterior views of the pelvis and hip, along with a cross-table lateral view to assess the acetabular component for signs of loosening, pelvic osteolysis, prominent intrapelvic screws, and retained cement [1]. The position and depth of the acetabular component, as well as the horizontal hip offset, should be noted. The cross-table or lateral radiograph is particularly useful for demonstrating the prominence of the anterior aspect of the acetabular component relative to the anterior rim of the bony acetabulum [17].

CT scans are valuable for measuring the version of the acetabular component and detecting anterior overhang, as well as iliopsoas tendon or bursal hypertrophy. They are especially helpful when body habitus or other factors make radiograph interpretation challenging. Studies have shown that significant acetabular component prominence on CT is associated with anterior iliopsoas impingement syndrome [5].

While MRI is less commonly used due to signal quality issues around prosthetic joints, advancements in software algorithms have improved its utility in evaluating pelvic osteolysis and periprosthetic soft tissues [1]. Specifically, the development of Multi-Acquisition Variable-Resonance Image Combination (MAVRIC) sequences has significantly reduced metal artifacts at 3 Tesla (3T) MRI, enhancing the visualization of the iliopsoas tendon, hip capsule, and other periprosthetic structures, thereby improving diagnostic confidence for detecting causes of groin pain such as iliopsoas impingement [18]. Ultrasonography, in the hands of an experienced radiologist, offers excellent soft-tissue contrast and can reveal anterior and medial displacement of the iliopsoas tendon by the acetabular component [19]. It is also used for guiding peritendinous corticosteroid injections for both diagnostic and therapeutic purposes [14].

The most frequently reported technique for evaluating anterior iliopsoas impingement and tendinitis is an image-guided diagnostic injection of contrast material into the iliopsoas tendon sheath [15]. This can be done using fluoroscopy, ultrasonography, or CT to guide needle placement. Injecting local anesthetic, alone or with a corticosteroid, can provide temporary pain relief, indicating the iliopsoas tendon as the pain source [1]. It is crucial that the injection targets only the tendon sheath and not the joint itself as that could give conflicting results [2].

Recent advancements in robotic-assisted THA have significantly improved the precision of acetabular component placement. Robotic systems, by ensuring more accurate restoration of the center of rotation and optimally combined anteversion, may reduce the risk of mechanical irritation to the iliopsoas tendon postoperatively [20]. Although direct evidence on iliopsoas impingement rates following robotic THA is limited, the enhanced control over cup positioning provided by robotic technology suggests a promising avenue for minimizing this complication.

Management

Management options for iliopsoas impingement following THA include conservative treatment, therapeutic injections, and surgical intervention.

Conservative Management

Nonsurgical management of acute or chronic iliopsoas tendinitis in the non arthroplasty patient includes rest, analgesics, nonsteroidal anti-inflammatory drugs, and physical therapy. Such symptomatic management should be attempted in the arthroplasty patient, as well [1]. The conservative management of iliopsoas impingement post-total hip replacement typically includes rest, stretching, and strengthening exercises, along with oral anti-inflammatory medications and local physiotherapy [8]. Stretching exercises, particularly those involving hip extension, are generally effective over a period of six to eight weeks [21]. Physiotherapy may include assisted extension and ultrasound therapy. Strengthening exercises focus on improving hip rotation strength, which often correlates with symptom reduction. These exercises are initially performed with the hip and knee flexed at 90°, using an elastic strap for resistance, and later progressing to a sideways prone position for outward rotation [8]. The regimen starts with daily exercises for the first four weeks, then reduces to three to four sessions per week until symptoms resolve. Additionally, daily stretching exercises for the hip flexors, buttocks, quadriceps, and hamstrings are included. Gait retraining, which involves maintaining hip stability by contracting the deep gluteal muscles, is also an essential part of the treatment protocol [8].

Injections

Corticosteroid injections into the iliopsoas bursa or tendon are a treatment option for iliopsoas impingement post-THA. In several case studies, injections were administered immediately after confirming the diagnosis of iliopsoas bursitis. These injections often provided temporary symptomatic relief, lasting from two to eight months, although symptoms sometimes recurred, leading to surgical intervention [21,22]. Imaging techniques are typically used to localize the iliopsoas bursa or tendon before injection, although some approaches do not require prior imaging [23]. Complications from the procedure are uncommon, but patients are advised of potential risks, including femoral nerve anesthesia and temporary quadriceps weakness [8]. These injections can offer temporary or even permanent relief, allowing for hip muscle retraining and potentially delaying or avoiding surgery.

Nonoperative treatments for anterior iliopsoas impingement and tendinitis have shown mixed success. A literature review reported that only 15 of 38 hips (39%) responded successfully to nonsurgical management [1,5,13,15,17,19]. A study of 11 patients with THA found that nine experienced at least 50% pain relief for up to a year following one or two sonography-guided injections [24]. Similarly, botulinum toxin type A injections into three lumbar sites of the psoas muscle provided temporary relief, though severe pain recurred within six months [25]. In contrast, a larger study involving 30 hips in 29 patients found that corticosteroid injections into the iliopsoas tendon sheath offered only short-term relief, with nonsurgical treatment failing in eight cases [5]. Another series of nine patients reported that only two were successfully treated without surgery, one after a single injection and another after two [26]. While local anesthetic and corticosteroid injections can help diagnose the condition and provide temporary symptom relief, their long-term effectiveness remains inconsistent. Consequently, the limited success of nonoperative approaches should be carefully weighed when discussing treatment options with patients.

Surgical Management

Surgical management of iliopsoas impingement has shown a high success rate, with various techniques available depending on the underlying cause. Iliopsoas tendon release or resection is a straightforward approach, typically chosen when imaging does not indicate anterior overhang of the acetabular component. This procedure can be performed through open, percutaneous, or arthroscopic techniques. In cases where the anterior overhang is present, acetabular revision is recommended to reposition the component correctly below the bony acetabular rim [7]. If impingement results from protruding cement or screws, their removal is necessary, often combined with iliopsoas tenotomy or resection. A review of the literature found that surgical intervention achieved a 91.5% success rate (65 out of 71 hips), with positive outcomes observed at an average follow-up of 22.7 months [1].

Iliopsoas tenotomy is typically the preferred and most straightforward procedure when imaging does not indicate anterior acetabular overhang [1]. Iliopsoas tenotomy can be performed using either open or arthroscopic methods, both of which have demonstrated comparable effectiveness. The surgical approach for tenotomy is generally dictated by the initial THA approach. A posterior approach technique includes making a 4-cm incision extending from the tip of the greater trochanter to the vastus tubercle. After dividing the deep fascia, the femur is internally rotated, allowing electrocautery to release the quadratus femoris muscle and expose the lesser trochanter. The iliopsoas tendon is then identified by palpation and severed using electrocautery [13]. An alternative approach is the anterolateral or direct lateral technique, which involves mobilizing the rectus and iliocapsularis muscles from the hip capsule. This provides direct access to the iliopsoas tendon, which is then transected near the lesser trochanter to reduce the risk of bleeding [5]. The success rates for open tenotomy are 81.0% via the posterior approach and 83.3% using the anterolateral or direct lateral approach [5,13,14].

In contrast, arthroscopic tenotomy is performed with the patient in a supine position, using traction to achieve capsular distention. Anterolateral and midanterior portals are created to facilitate a transcapsular release of the iliopsoas tendon with radiofrequency probes while preserving surrounding musculature [27,28]. In some cases, an extra-articular approach near the lesser trochanter is used to minimize the risk of prosthetic component damage. Notably, arthroscopic procedures have reported no complications, whereas open tenotomy studies have documented a 33.3% complication rate, including heterotopic ossification and trochanteric bursitis [5]. 

When preoperative radiographs or CT scans reveal anterior acetabular component overhang, acetabular revision is recommended. The revision procedure generally follows the same approach as the initial arthroplasty. If intraoperative findings reveal inflammation or thickening of the iliopsoas tendon or bursa, resection is recommended [1]. The revision procedure involves removing the existing acetabular component, medializing the new component, and increasing anteversion to position the anterior edge below the bony acetabular rim, thereby reducing impingement [4,17]. Studies have suggested that acetabular revision without greater trochanteric osteotomy or reinforcement rings results in lower complication rates and improved outcomes [5]. For cases where impingement is caused by protruding anterior cement or excessively long screws, these must be directly addressed along with iliopsoas tenotomy or resection [4].

## Conclusions

Iliopsoas impingement is an important and often overlooked cause of groin pain following THA. Due to its nonspecific symptoms and overlap with other pathologies, diagnosis requires careful clinical assessment, supported by targeted imaging and diagnostic injections. While conservative treatments and corticosteroid injections may provide temporary symptom relief, surgical management, particularly iliopsoas tenotomy or acetabular revision in the presence of component overhang, offers more definitive and lasting results. Recognizing this condition early and adopting a structured diagnostic and treatment approach is critical to improving patient outcomes and reducing unnecessary delays in care. These efforts are further supported by the growing experience of surgeons and the increasing adoption of robotic-assisted total hip replacement procedures, which may contribute to more consistent results.
